# Editorial: Immunometabolism and therapeutic targeting of aggressive cancers

**DOI:** 10.3389/fonc.2023.1226078

**Published:** 2023-06-12

**Authors:** Maša Ždralević, Jacques Pouysségur

**Affiliations:** ^1^ Institute for Advanced Studies, University of Montenegro, Podgorica, Montenegro; ^2^ Medical Biology Department, Centre Scientifique de Monaco (CSM), Monaco, Monaco; ^3^ Centre A. Lacassagne, Institute for Research on Cancer and Aging (IRCAN), Université Côte d'Azur, CNRS, INSERM, Nice, France

**Keywords:** immunometabolism, cancer metabolism, immune checkpoint signaling, immunotherapy, combinational therapy

Research on the two emerging hallmarks of cancer, reprogramming of cellular energy metabolism and immune system evasion, has gained momentum in the last decade and opened new avenues for cancer management. Complex metabolic changes that occur in cancer cells during tumorigenesis are well-documented, whereas immunometabolism is an emerging field that focuses on the metabolic modulation of immune cells as an important determinant of their phenotype and function. Despite impressive advances in the field of cancer immunotherapy, our understanding of metabolic interactions of cancer and immune cells in the context of both tumor microenvironment (TME) as well as immunotherapy is limited.

The main problem we addressed in this Research Topic is how a better understanding of metabolic reprogramming of both cancer and immune cells, their interaction, and the use of metabolism-targeting drugs, could improve current cancer immunotherapy and its synergy with chemo-, radio-, and targeted therapy. Many recent findings demonstrated that the metabolic modulation of myeloid, CD8^+^, T, and natural killer (NK) cells are instrumental in overcoming immune suppression and enhancing therapeutic efficiencies, such as adoptive cell therapy and chimeric antigen receptor-engineered T-cell therapy. Furthermore, accumulating evidence suggests that the activation of immune checkpoint signaling can influence the metabolic profile of both cancer and immune cells. Therefore, a better and more precise understanding of metabolic interactions between cancer and immune cells is necessary for the development of new combinatorial therapies and better treatment outcomes.

The scope of this Research Topic was to unravel the complexities of the interplay between metabolic regulation and the immune response in cancer and their implications on therapeutic targeting and overcoming therapy resistance. Specific themes to be addressed, but are not limited to, were:

1) The role of metabolism in immune cell activation and differentiation
2) The mechanisms of metabolic interactions in TME
3) The therapeutic targeting of metabolic vulnerabilities
4) The implications of cancer and immune cells’ metabolism on immunotherapy
5) Combinatorial strategies with conventional therapies for the optimization of cancer management.

Slattery et al. investigated the metabolic fitness of circulating NK cells, responsible for direct cytotoxicity toward cancer cells (1), and known to undergo severe metabolic dysregulation in older patients (2). Exhaustion of NK cells reported in many cancer types (3) poses a significant problem to NK cell immunotherapy. Slattery et al. found that in pediatric patients newly diagnosed with neuroblastoma, prior to any medical treatment, NK cells had an activated phenotype and a high expression of CD16, a key receptor mediating therapeutic antibody-dependent cellular cytotoxicity (ADCC). These NK cells maintained the ability to respond to cytokines; IFNγ production was impaired, but granzyme B upregulation was significant. Regarding their metabolic profile, NK cells from patients with neuroblastoma had higher glycolytic flux and mTORC1 signaling activity, compared to healthy controls. Furthermore, NK cells from patients had significantly higher mitochondrial ROS levels, increased ATP5B protein levels, and punctate mitochondrial structure, pointing to the onset of mitochondrial dysfunction in these cells. Finally, fermentative glycolysis was proven to be the primary bioenergetic pathway required for NK cell killing by ADCC. These results suggest that in patients with neuroblastoma, prior to any intervention, NK cells are primed by cancer itself to increase glycolysis, facilitating ADCC. This finding is potentially relevant for the clinical setting since these autologous circulating NK cells could be harvested before the therapeutic treatment to be used for adjuvant immunotherapy instead of depending on NK cells to regenerate post-treatment.

Metabolism has been proposed to affect the response to chemo- and immunotherapy (4, 5). In their work with patients with advanced non-small cell lung cancer (NSCLC), Mei et al. performed an untargeted metabolomic analysis in order to identify potential predictive biomarkers for the efficiency of chemoimmunotherapy as the first-line therapy. They have identified seven metabolites, including pyruvate, threonine, alanine, urea, oxalate, elaidic acid, and glutamate as the key metabolites (AUC was 0.86 for the combined biomarker model) in correlation with the poor response to therapy. These metabolites are involved in the urea cycle, glucose-alanine cycle, glycine and serine metabolism, alanine metabolism, and glutamate metabolism, known to be involved in cancer progression. Metabolic profiling could therefore be proposed as a complementary strategy for determining the best personalized approach to NSCLC treatment.

In a mini-review, Pajai et al. summarized key aspects of metabolic reprogramming of cancer and immune cells, as well as main therapeutic approaches targeting carbohydrate, amino acid, and fatty acid metabolism. They emphasized the crosstalk between cancer and immune cells at the level of metabolism and the possibilities of targeting metabolic vulnerabilities.

The development of immune checkpoint inhibition, such as PD-1 receptor and its associated ligand PD-L1, has been one of the major breakthroughs in the field of cancer immunotherapy (6). However, only a limited number of patients respond adequately to therapy, and there is a need for new predictive biomarkers in order to overcome immune escape and resistance to treatment (7). Babl et al. have shown that low-density lipoprotein (LDL) balanced CD4^+^ and CD8^+^ T cells metabolism and induced a central memory phenotype in CD4^+^ cells. Upon stimulation with IL-2, LDL significantly up-regulated CD154 (CD40L), a co-stimulatory marker, in the CD4^+^ T cell fraction, but, overall, LDL was associated with the less exhausted phenotype in both subtypes of T cells. This has led to an enhanced anti-tumor response in a spheroid co-culture model. Pre-activated T-cells co-cultured with colon carcinoma HCT116 spheroids revealed that a combination of LDL with an anti-PD-1 antibody induced a significant reduction of spheroid viability.

The tumor microenvironment is characterized by glutamine deficiency, which affects all immune cell subtypes and their immunoregulatory pathways (8). Schoeppe et al. investigated the effects of glutamine deficiency on human myeloid cells, especially dendritic cells (DC). By using the monocytic leukemia cell line THP-1 as a model, indeed, they showed that human monocytes depend on exogenous glutamine for proliferation. However, they adapt to glutamine-free conditions by up-regulating glutamine synthase, the enzyme that catalyzes *de novo* glutamine synthesis. Furthermore, glutamine synthase was proven to be essential for monocyte differentiation into DCs and macrophages in the absence of glutamine; therefore, targeting the metabolic crosstalk between myeloid and tumor cells could represent an attractive treatment strategy.

## Author contributions

All authors listed have made a substantial, direct, and intellectual contribution to the work and approved it for publication.
